# Supplementary material for: Low maternal vitamin D is associated with increased risk of congenital and peri/postnatal transmission of Cytomegalovirus in women with HIV
Source: PLoS One. 2020 Feb 13;15(2):e0228900. doi: 10.1371/journal.pone.0228900 (PMC7018030; doi:10.1371/journal.pone.0228900)
Supplement: S1 Table — (DOCX) [file pone.0228900.s001.docx]

| **S1 Table. Number of positive CMV tests by test type for All CMV+ infants** | | | |
| --- | --- | --- | --- |
|  | **Number of CMV+ tests** | | |
|  | **Urine culture** | **Oral culture** | **Blood PCR** |
| **Congenital CMV+ infants:** |  |  |  |
| Infant 1 | 1 | 1 | 1 |
| Infant 2 | 1 | 1 | 1 |
| Infant 3 | 2 | 2 | 1 |
| Infant 4 | 2 | 3 | 1 |
| Infant 5 | 3 | 2 | 1 |
| Infant 6 | 3 | 4 | 1 |
| Infant 7 | 3 |  | 1 |
| Infant 8 | 3 |  | 1 |
| Infant 9 |  |  | 1 |
| Infant 10 |  |  | 2 |
| Infant 11 | 1 |  |  |
| Infant 12 | 1 |  |  |
| Infant 13 | 4 |  |  |
| Infant 14 |  | 2 |  |
|  |  |  |  |
| **Peri/postnatal CMV+ infants:** |  |  |  |
| Infant 1 | 2 | 2 | 1 |
| Infant 2 | 1 | 1 |  |
| Infant 3 | 1 | 2 |  |
| Infant 4 | 1 | 2 |  |
| Infant 5 | 1 |  |  |
| Infant 6 | 1 |  |  |
| Infant 7 | 1 |  |  |
| Infant 8 | 1 |  |  |
| Infant 9 | 1 |  |  |
| Infant 10 | 1 |  |  |
| Infant 11 | 1 |  |  |
| Infant 12 | 1 |  |  |
| Infant 13 | 1 |  |  |
| Infant 14 | 1 |  |  |
| Infant 15 | 1 |  |  |
| Infant 16 | 1 |  |  |
| Infant 17 | 1 |  |  |
| Infant 18 | 2 | 1 |  |
| Infant 19 | 2 | 1 |  |
| Infant 20 | 2 |  |  |
| Infant 21 | 2 |  |  |
| Infant 22 |  | 1 |  |
| Infant 23 |  | 2 |  |
| Infant 24 |  | 2 |  |
