# Supplementary material for: Low maternal vitamin D is associated with increased risk of congenital and peri/postnatal transmission of Cytomegalovirus in women with HIV
Source: PLoS One. 2020 Feb 13;15(2):e0228900. doi: 10.1371/journal.pone.0228900 (PMC7018030; doi:10.1371/journal.pone.0228900)
Supplement: S2 Table — (DOCX) [file pone.0228900.s002.docx]

| **S2 Table. 25-hydroxyvitamin D sufficiency by infant transmission category** | | | | | | |
| --- | --- | --- | --- | --- | --- | --- |
|  | | **Congenital CMV+ (N=14)** | | **Peri/postnatal CMV+ (N=24)** | **CMV negative (N=302)** | **Total sample (N=340)** |
| **25-hydroxyvitamin D ng/ml** | | |  | |  |  |
| ≤ 10 | 0 | | 0 | | 8 (2.6%) | 8 (2.3%) |
| 11 - 20 | 3 (21.4%) | | 5 (20.8%) | | 73 (24.2%) | 81 (23.8%) |
| 21 - 31 | 9 (64.3%) | | 9 (37.5%) | | 123 (40.7%) | 141 (41.5%) |
| 32+ | 2 (14.3%) | | 10 (41.7%) | | 98 (32.5%) | 110 (32.4%) |
