# Supplementary material for: Low maternal vitamin D is associated with increased risk of congenital and peri/postnatal transmission of Cytomegalovirus in women with HIV
Source: PLoS One. 2020 Feb 13;15(2):e0228900. doi: 10.1371/journal.pone.0228900 (PMC7018030; doi:10.1371/journal.pone.0228900)
Supplement: Attachment — Submitted filename: Response to Reviewers.pdf [file pone.0228900.s003.pdf]

December 17, 2019

Dear Dr. Nevels and the PLOS ONE editorial staff,

Thank you for your thoughtful consideration and suggested revisions of our recent submission to your journal, "Low maternal vitamin D is associated with increased risk of congenital and peri/postnatal transmission of Cytomegalovirus in women with HIV." Please find the explanations and rebuttals for the requested revisions or questions posed by the academic editor and / or reviewer outlined below.

1. Rationale for not screening the infant urine and saliva samples for CMV by PCR:
   This study was a retrospective study nested in a prospective natural history study. The infant samples were originally tested based on the protocol for the natural history study which entailed only CMV cultures. Unfortunately, there were not sufficient samples available to retest for CMV using PCR methodology. This may have underestimated the prevalence of CMV infections in the infants. This explanation has been added to the discussion section, in the paragraph describing study limitations.

2. Inclusion of a breakdown of samples which tested positive:
   In order to provide additional information regarding which tests were positive for infants categorized as CMV infected, a table was added as supplementary data, and cited in the manuscript in the first paragraph of the results section (**S1 Table. Number of positive CMV tests by test type for all CMV+ infants**)

3. Style requirements:
   Requirements have been reviewed and the revised document has incorporated all necessary changes.

4. Ethics statement:
   The ethics statement from the online submission was added to the manuscript at the end of the Methods section.

5. Data accessibility:
   We are unable to publicly share the dataset from this study due to ethical and legal restrictions. According the University of Southern California's Office of Ethics and Compliance, there is no way to make the dataset truly de-identified. The dataset is made up solely of women with a diagnosis of HIV and their offspring. The diagnosis of HIV is an indirect identifier and considered sensitive information requiring additional protections. Additional indirect identifiers in the dataset include age, race/ethnicity, and sex. Additionally, the consent signed by the study participants did not include a provision for their data to be made publicly accessible. Data requests can be directed to department administrator, Carlota Obnillas, obnillas@usc.edu, and if the request is approved, the USC Stevens Center for Innovation will assist and create a Data Transfer Agreement.

6. <u>Data not shown:</u>
Data to support the statements summarizing the 25-hydroxyvitamin D levels of the cohort, in the third paragraph of the results section, has been added as a supplementary table (**S2 Table 25-Hydroxyvitamin D sufficiency by infant transmission category**)

7. <u>Concern regarding the influence of storage time on vitamin D measurements:</u>
Vitamin D is established as very stable in frozen specimens and can be stored indefinitely according to several lab manuals or processing instructions. The levels also maintain stability in the face of multiple repeated freeze-thaw cycles as well. This information has been added to the manuscript in the methods section, in the paragraph on Vitamin D testing.
https://www.nationaljewish.org/for-professionals/diagnostic-testing/adx/tests/vitamin-d-25-hydroxy-total

Thank you again for the opportunity to revise our manuscript. Please let us know if there are any other questions or concerns regarding this work.

Sincerely,

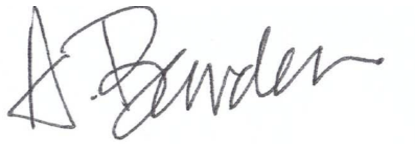

Allison Bearden, MD, MPH (Corresponding author)
Assistant Professor, Department of Pediatrics,
Division of Infectious Diseases
Keck School of Medicine of USC
1640 Marengo St. HRA 300
Los Angeles, Ca 90089-9033
Phone: 323 865-1585
Fax: 323 224-5013
Email: Allison.bearden@usc.edu

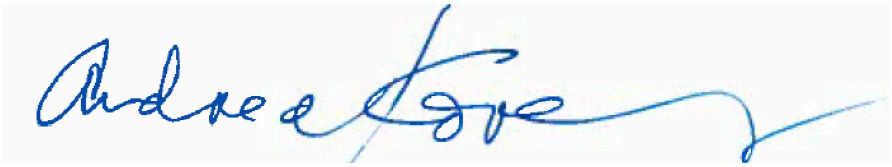

Andrea Kovacs, MD
Professor of Pediatrics and Pathology,
Director, Maternal, Child and Adolescent Center for
Infectious Diseases and Virology
Keck School of Medicine of USC
1640 Marengo St. HRA 300
Los Angeles, Ca 90089-9033
Phone: 323 865-1585
Fax: 323 224-5013
Email: akovacs@usc.edu
